# Prioritising pathogens for the management of severe febrile patients to improve clinical care in low- and middle-income countries

**DOI:** 10.1186/s12879-020-4834-1

**Published:** 2020-02-10

**Authors:** Jennifer Osborn, Teri Roberts, Ethan Guillen, Oscar Bernal, Paul Roddy, Stefano Ongarello, Armand Sprecher, Anne-Laure Page, Isabela Ribeiro, Erwan Piriou, Abiy Tamrat, Roberto de la Tour, V. Bhargavi Rao, Laurence Flevaud, Tomas Jensen, Lachlan McIver, Cassandra Kelly, Sabine Dittrich

**Affiliations:** 1 Foundation for Innovative New Diagnostic (FIND), Geneva, Switzerland; 2 Access Campaign, Médecins Sans Frontières, Geneva, Switzerland; 3 Médecins Sans Frontières, New York, USA; 4 Médecins Sans Frontières, Brussels, Belgium; 5 Epicentre, Paris, France; 6 Drugs for Neglected Diseases initiative (DNDi), Geneva, Switzerland; 7 Médecins Sans Frontières, Amsterdam, The Netherlands; 8 Médecins Sans Frontières, Geneva, Switzerland; 9 Médecins Sans Frontières – Manson Unit, London, UK; 10 Médecins Sans Frontières, Barcelona, Spain; 11 Nuffield Department of Medicine, University of Oxford, Oxford, UK

**Keywords:** Severe febrile illness, Disease prioritization, Fever causing pathogens, Diagnostics

## Abstract

**Background:**

Severe febrile illness without a known source (SFWS) is a challenge for clinicians when deciding how to manage a patient, particularly given the wide spectrum of potential aetiologies that contribute to fever. These infections are difficult to distinguish clinically, and accurate diagnosis requires a plethora of diagnostics including blood cultures, imaging techniques, molecular or serological tests, and more. When laboratory services are available, a limited test menu hinders clinical decision-making and antimicrobial stewardship, leading to empiric treatment and suboptimal patient outcomes. To specifically address SFWS, this work aimed to identify priority pathogens for a globally applicable panel for fever causing pathogens.

**Method:**

A pragmatic two-pronged approach combining currently available scientific data in an analytical hierarchy process and systematically gathered expert input, was designed to address the lack of comprehensive global aetiology data. The expert re-ranked list was then further adapted for a specific use case to focus on community acquired infections in whole blood specimens. The resulting list was further analysed to address different geographical regions (Asia, Africa, and Latin America), and Cohen kappa scores of agreement were calculated.

**Results:**

The expert ranked prioritized pathogen list generated as part of this two-pronged approach included typhoidal *Salmonella*, *Plasmodium* species and *Mycobacterium tuberculosis* as the top 3 pathogens. This pathogen list was then further adapted for the SFWS use case to develop a final pathogen list to inform product development. Subsequent analysis comparing the relevance of the SFWS pathogen list to multiple populations and geographical regions showed that the SFWS prioritized list had considerable utility across Africa and Asia, but less so for Latin America. In addition, the list showed high levels of agreement across different patient sub-populations, but lower relevance for neonates and symptomatic HIV patients.

**Conclusion:**

This work highlighted once again the challenges of prioritising in global health, but it also shows that taking a two-pronged approach, combining available prevalence data with expert input, can result in a broadly applicable priority list. This comprehensive utility is particularly important in the context of product development, where a sufficient market size is essential to achieve a sustainable commercialized diagnostic product to address SFWS.

## Background

Severe febrile illness without a known source (SFWS) is a challenge for clinicians when deciding how to manage a patient, particularly given the wide spectrum of potential aetiologies, including bacterial and fungal bloodstream infections, bacterial zoonoses, malaria, and viral infections [1, 2]. These infections can be difficult to distinguish clinically; accurate diagnosis relies on timely use and interpretation of diagnostics including blood cultures, imaging techniques, molecular or serological tests, and more [3]. In low- and middle-income countries (LMICs), fever is the primary reason for seeking health care, and mortality rates for patients requiring hospital admission for severe febrile illness (SFI) range from approximately 5 to 20% [1–6]. In general, diagnostic capacity in LMICs is insufficient, even at the hospital level [3]. Thus, management decisions for patients presenting with SFI are primarily based on clinical assessment algorithms, which often lead to misdiagnoses due to poor diagnostic specificity and empiric treatment [7]. Often this results in unnecessary antibiotics for viral infections or polytherapy where a multitude of antimicrobials are given at the same time to cover all the bases. To inform clinical management and improve clinical outcomes for SFWS, it is crucial that diagnostic test results are both timely and informative [3, 6].

Rapid and accurate diagnostic tests that can be implemented in resource-limited settings (RLS) could have substantial positive impact on the management of febrile illness, both severe and non-severe.

As a result of increased malaria elimination efforts and the subsequent worldwide decline in malaria cases, clinicians in LMICs are now encountering an increasing number of malaria rapid diagnostic test (RDT)-negative febrile patients and/or malaria RDT-positive febrile patients who are co-infected with both *Plasmodium* as well as another fever-inducing pathogen [8–11]. To date, in LMICs, beyond the use of a malaria RDT and/or microscopy for some locations [12], no aetiology-based diagnostic test is currently employed or recommended by the World Health Organization (WHO) for any other fever-inducing pathogen. However, given the large number of cases and the benefit to individual and public health that results from targeted and effective treatment administration as opposed to undifferentiated empiric treatment, a push towards integrated disease and febrile clinical management is gaining momentum [13–16].

Recent advances in diagnostics have resulted in the development of cartridge-based diagnostic platforms capable of simultaneously detecting a number of pathogens [17–19]. However, due to a dearth in aetiology data spanning all relevant demographics, regions, and seasons [1], the identification, ranking, and prioritization of pathogens for inclusion in a SFWS cartridge syndromic panel is fraught with challenges. To date, there are limited available studies that have measured pathogen positivity rates and thus, both regional and global pathogen prioritization rankings are difficult to match to particular demographics or geography [4, 20].

As part of a wider initiative lead by Médecins Sans Frontières (MSF), the WHO, and the Foundation of Innovative New Diagnostics (FIND), the parameters of a multi-pathogen and multi-analyte diagnostic platform (MAPDx) to support improved diagnostic capacity at level 2 or above [21, 22] were defined. This work aimed to prioritize common fever-causing pathogens to inform pathogens for use on a fever-specific diagnostic cartridge for diagnosis in LMICs using a two-pronged approach combining a) currently available scientific data incorporated into an analytical hierarchy process (AHP), and b) systematically gathered expert input to compensate for the paucity of regional and global pathogen-specific data availability. This work defined a general fever priority pathogen list through this two-pronged approach that aimed to bridge ecological and transmission variability. This general priority list was then further adapted to the specific use case of SFWS to define a set of priority fever-causing pathogens for the MAPDx platform [23] and demonstrates how the general fever priority pathogen list could be used as a starting framework for various other applications.

## Methods

A multifaceted quantitative and qualitative approach was designed and implemented to generate informative data where there is otherwise a dearth of comprehensive global aetiology data for SFWS in LMICs. The approach comprised a data-derived Analytical Hierarchy Process [24] process followed by systematically gathered expert input. Due to non-comparable case estimates (e.g. employment of varying inclusion/exclusion criteria and diagnostic testing algorithms) across studies identifying pathogens that cause SFI, an available systematic review by Prasad et al. was employed, which compared a set number of SFI causes across settings [1].

### Data-derived approach using an AHP process

AHP is structured method used to organise and analyse complex decision process. It combines mathematics and psychology and was developed in the 1970 and subsequently used to support public health prioritisation [25, 26].

#### Priority profiling, pairwise comparison and ranking

**Fig. 1:**
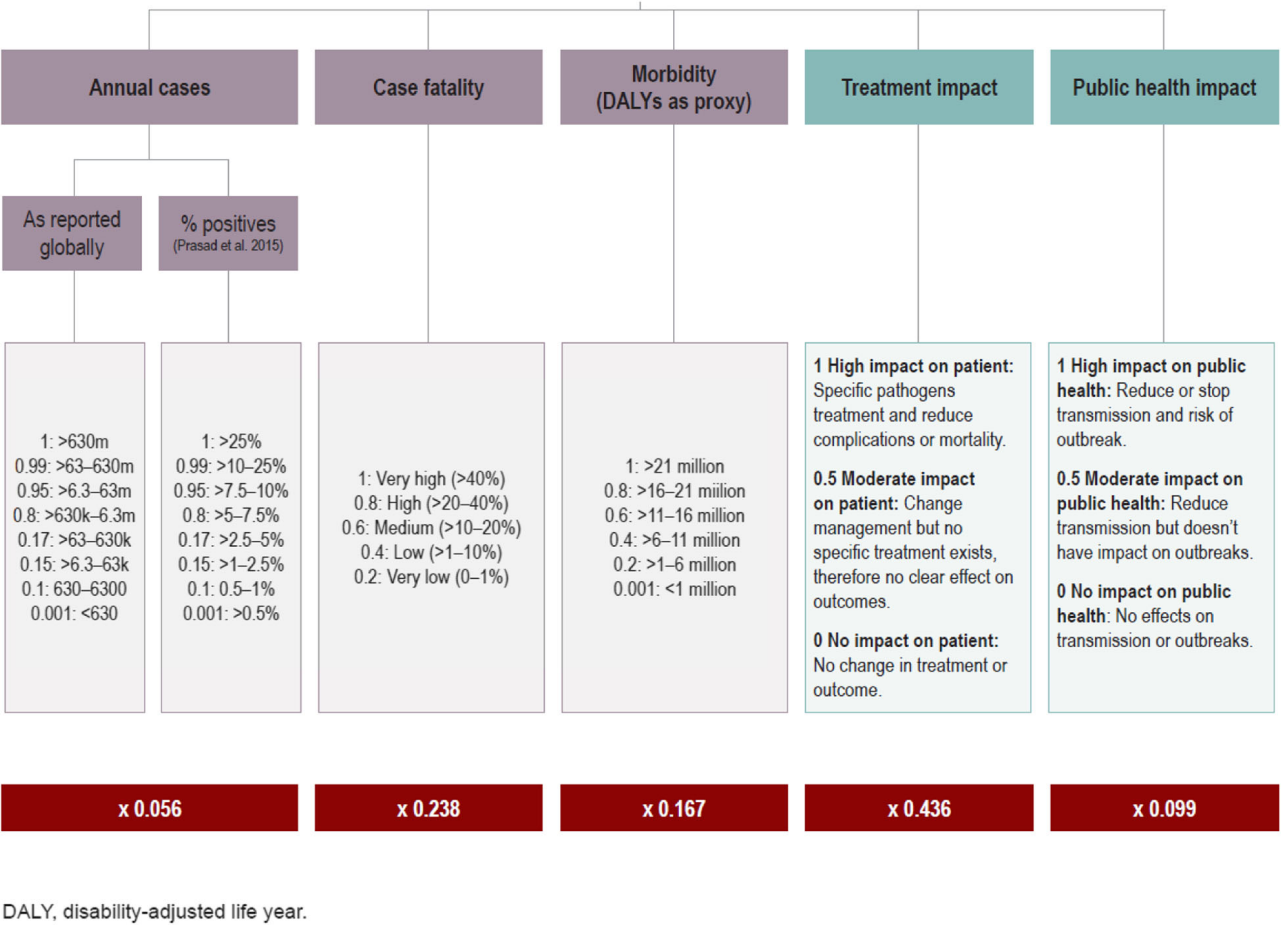
Hierarchical structure: analytical hierarchy process (AHP) prioritization model including weights to prioritze pathogens for severe febrile illness diagnostics

To prioritize the list of pathogens presented by Prasad et al., five criteria and corresponding sub-criteria for evaluating a pathogen’s severity and diagnostic impact were identified based on previously published pathogen prioritization efforts [26, 27] (Fig. 1). Based on a review of related literature [24, 28], the following criteria were determined to be relevant for the current use case: number of annual cases (as reported globally and positivity rates reported by Prasad et al.*)*; case fatality rate; morbidity using disease-adjusted life-years [29]; and impact on treatment decisions (measured from high to low depending on treatment available for a specific pathogen and the diagnostic potential to reduce complications and/or death). Each criterion had defined sub-criteria, as presented in Fig. 1 and was informed by a targeted literature review (Additional file 2). In brief, annual cases and DALYs were taken from specific publications [1, 29]; mortality rate was calculated from multiple publications (Additional file 1) and the clinical and public health impact was assessed and assigned by a subset of authors (OB, ALP, RDLT, EP, AS, IR, TJ, AT, LM) and additional MSF experts. In order to enable a more granular scoring, values or ranges for all sub-criteria were assigned a relative value within the criteria to reflect the impact on the overall scoring; a linear scale was chosen for “mortality” and “morbidity”, and a sigmoid-like scale for “annual cases” (Fig. 1). Subsequently, the associated weights were determined using the AHP absolute evaluation method performed by a subset of the authors with a clinical or public health background (*n* = 9; OB, ALP, RDLT, EP, AS, IR, TJ, AT, LM) using a dedicated pairwise comparison tool (AHP Process, K.D Goepel Version 11.10.2017, Additional file 3**)**. Next, the annual case sub-criteria were individually multiplied by the weight and then added up. All sub-criteria were then multiplied by the criteria’s weight. All pathogen-specific criterion scores were summed and ranked accordingly (Additional file 1).

#### Sensitivity analysis

A sensitivity analysis was performed to determine whether weightings or categories were unduly driving the ranking, and to confirm robustness of the model. Random noise was added to the values for each criterion used in varying amounts (up to 50% of the possible range for each category). In addition to the absolute score, the percentage change in score and the width of the 95% confidence interval were evaluated. The process was repeated 10,000 times and bootstrapped estimates of the scores were derived for each pathogen [30]. Analyses were performed using R version 3.4.3.

### Expert re-ranking

An online qualitative survey was developed to allow for re-ranking of the AHP-derived pathogen list for detection of SFWS for the general population in a whole blood specimen to inform clinical action. The purpose of the survey was to gather expert opinions in the pathogen prioritization process to account for possible underrepresentation in the literature and clinical relevance. The survey (Additional file 2) was sent to relevant global stakeholders with request for their experience- and opinion-based feedback. Stakeholders were identified from relevant publications, national and international committees, known relevant academic research groups, and international organizations operating in this space. The experts were requested to re-rank the AHP-derived pathogen list that resulted from the AHP ranking (the top 32 pathogens were provided). To enable an informed decision by the stakeholders, the use-case, purpose and diagnostic aim of the work was clearly stated in the introductory documents (Additional file 4). In addition to re-ordering the pathogen priority list to the survey respondents’ preferences, respondents were allowed to remove or add priority pathogens to their individual rankings (i.e. wildcards). The survey tool, Survey Gizmo, automatically calculated a score for rank distribution. This score was a weighted calculation based on the total number of pathogens (32) respondents were able to rank. The final score per pathogen was the sum of all the weighted values. Data from survey respondents was used to compile a general fever priority pathogen list that incorporated all survey participants’ rankings, removals and additions.

### Severe fever of unknown source use case specific list

To develop a list of pathogens for a fever panel for use in a LMIC district-hospital setting as part of a larger initiative by MSF, the resulting general fever priority pathogen list (specific to this “MSF-use case”) that was the output of the two-pronged approach (Table 1) was further refined for relevance to the use case for testing of a single blood specimen for individual patient management of SFWS and to account for existing testing algorithms at public facilities (e.g. using malaria rapid diagnostic tests, Xpert MTB/RIF (Cepheid), Cryptococcal antigen lateral flow assay (Immy)). In addition, focus was given to community-acquired versus nosocomial infections to support primary management decisions. The resulting SFWS priority pathogens formed the fever panel to be run on the MAPDx platform (Table 2), where the top 10 pathogens were included in a minimally required set and where, in order of priority, > 10 pathogens were listed as the optimal fever pathogen panel.

**Table 1:** Pathogen rankings following the data-driven AHP approach and the expert re-ranking. AHP, analytical hierarchy process; spp., species. *Serogroups A, B, C, W-135, Y, and X; †Types A, B and C; ‡Types 1, 2 and 3

	AHP-derived list	General fever priority pathogen list	
Rank	Pathogen	Pathogen	Change in rank
1	*Plasmodium* spp	Typhoidal *Salmonella*	↑
2	*Cryptococcus* spp.*	*Plasmodium* spp	↓
3	*Mycobacteria tuberculosis*	*Mycobacterium tuberculosis*	no change
4	*Mycobacterium avium* complex (MAC)	*Streptococcus pneumoniae*	↑
5	*Klebsiella* spp	*Staphylococcus aureus*	↑
6	*Neisseria meningitidis* (serogroups A, B, C, W-135, Y, and X)	Non-typhoidal *Salmonella*	↑
7	*Shigella* spp	*Escherichia coli*	↑
8	*Burkholderia pseudomallei*	*Neisseria meningitidis* (serogroups A, B, C, W-135, Y, and X)	↓
9	*Streptococcus pneuomiae*	*Rickettsia* spp	↑
10	*Orientia tsutsugamushi*	*Klebsiella* spp	↓
11	Typhoidal *Salmonella*	*Leptospira* spp.	↑
12	*Haemophilus influenzae*	*Cryptococcus* spp	↓
13	*Pseudomonas* spp	*Brucella* spp	↑
14	*Acinetobacter baumannii*	*Shigella* spp	↓
15	*Rickettsia* spp	*Orientia tsutsugamushi*	↓
16	*Leptospira* spp	*Haemophilus influenzae*	↓
17	*Escherichia coli*	*Burkholderia pseudomallei*	↓
18	*Staphylococcus aureus*	*Pseudomonas* spp.	↓
19	*Brucella* spp	*Mycobacterium avium* complex (MAC)	↓
20	Non-typhoidal *Salmonella*	*Acinetobacter baumannii*	↓
21	*Histoplasma capsulatum*	Influenza virus A, B, and C	↑
22	*Coxiella burnetii*	Dengue virus 1, 2, and 3	↑
23	Proteus mirabilis*	*Coxiella burnetii*	↓
24	*Enterobacter* spp	*Histoplasma capsulatum*	↓
25	*Citrobacter* spp.*	*Leishmania donovani/ infantum*	New
26	Influenza virus A, B, and C	Group B *Streptococcus*^+^	New
27	*Borrelia recurrentis*	Lassa fever^+^	New
28	Japanese encephalitis virus*	*Enterococcus faecalis*^+^	New
29	Yellow fever virus*	*Enterobacter* spp.^+^	New
30	West Nile virus*	*Borrelia recurrentis*	↓
31	Dengue virus 1, 2, and 3	Chikungunya virus	↓
32	Chikungunya virus		

* Removals based on expert opinion

+Additions based on expert opinion

**Table 2:** Pathogen ranking for severe febrile illness without a known source (SFWS) use case

Rank	Pathogen	Primarily a community-acquired or nosocomial pathogen
1	Typhoidal *Salmonella*	Community-acquired
2	*Streptococcus pneumoniae*	Community-acquired
3	*Staphylococcus aureus*	Community-acquired
4	Non-typhoidal *Salmonella*	Community-acquired
5	*Escherichia coli*	Community-acquired
6	*Rickettsia* spp	Community-acquired
7	*Leptospira* spp.	Community-acquired
8	*Brucella* spp	Community-acquired
9	*Burkholderia pseudomallei*	Community-acquired
10	*Coxiella burnetii*	Community-acquired
11	*Neisseria meningitidis*	Community-acquired
12	*Klebsiella* spp	Community-acquired and nosocomial
13	*Orientia tsutsugamushi*	Community-acquired
14	*Haemophilus influenzae*	Community-acquired
15	Dengue virus 1, 2, and 3	Community-acquired
16	*Histoplasma capsulatum*	Community-acquired
17	Lassa fever	Community-acquired
18	*Enterococcus faecalis*	Community-acquired
19	*Borrelia recurrentis*	Community-acquired
20	Chikungunya virus	Community-acquired
21	*Pseudomonas* spp	Nosocomial
22	*Acinetobacter baumannii*	Nosocomial
23	*Enterobacter* spp	Nosocomial

Further blood was prioritized as a testing matrix above other clinical specimens. In addition, the SFWS specific use case list was evaluated against the following important sub-populations: paediatrics (age groups: 0 days to < 1 month; ≥1 month to 4.9 years, ≥5 years to 15 years); individuals with symptomatic human immunodeficiency virus (HIV) infection; and the geographic regions of Africa, Asia, or Latin America. Structured literature searches using MEDLINE/PubMed and Cochrane Library with MeSH terms were conducted to assess relevance. A pathogen was considered to be relevant to a specific subpopulation if at least one peer-reviewed scientific publication could be identified that established a pathogen as being present in any of the investigated sub-populations or regions. Applicability of the pathogen list was assessed by calculating the percentage of agreement between subpopulations and Cohen’s Kappa score (0.01–0.20 as none to slight, 0.21–0.40 as fair, 0.41–0.60 as moderate, 0.61–0.80 as substantial, and 0.81–1.00 as almost perfect agreement).

## Results

### AHP process

The preliminary pathogen ranking (AHP-derived list) using available aetiology data developed following the application of the AHP process is shown in Table 1. Malaria (*Plasmodium* species) was ranked as the highest priority (weighted score percentage 95.8%); other pathogens in the top five were *Cryptococcus* species (86.4%), *Mycobacterium tuberculosis* (86.4%), *Mycobacterium avium* complex (85.6%), and *Klebsiella* species (77.5%).

The sensitivity analysis demonstrated that treatment impact was the most critical variable. Changes in treatment impact affected the scoring of several pathogens, in particular of those that originally had high values for this variable. However, the magnitude of change was small (usually less than 10% of the original score). Patient impact also affected the uncertainty of the estimates: changes of 50% in the source data resulted in an increase of up to 20% in 95% confidence interval-widths. For the majority of pathogens, severity of disease was the second driving factor for uncertainty after treatment impact, followed by burden of disease. The remaining variables had a limited impact, mostly due to their small contribution to the overall score. While the values of the scores were modified by changes in these variables, modifications were generally consistent across pathogens, thus the relative ranking of pathogens was only minutely affected.

### Expert re-ranking process

**Fig. 2:**
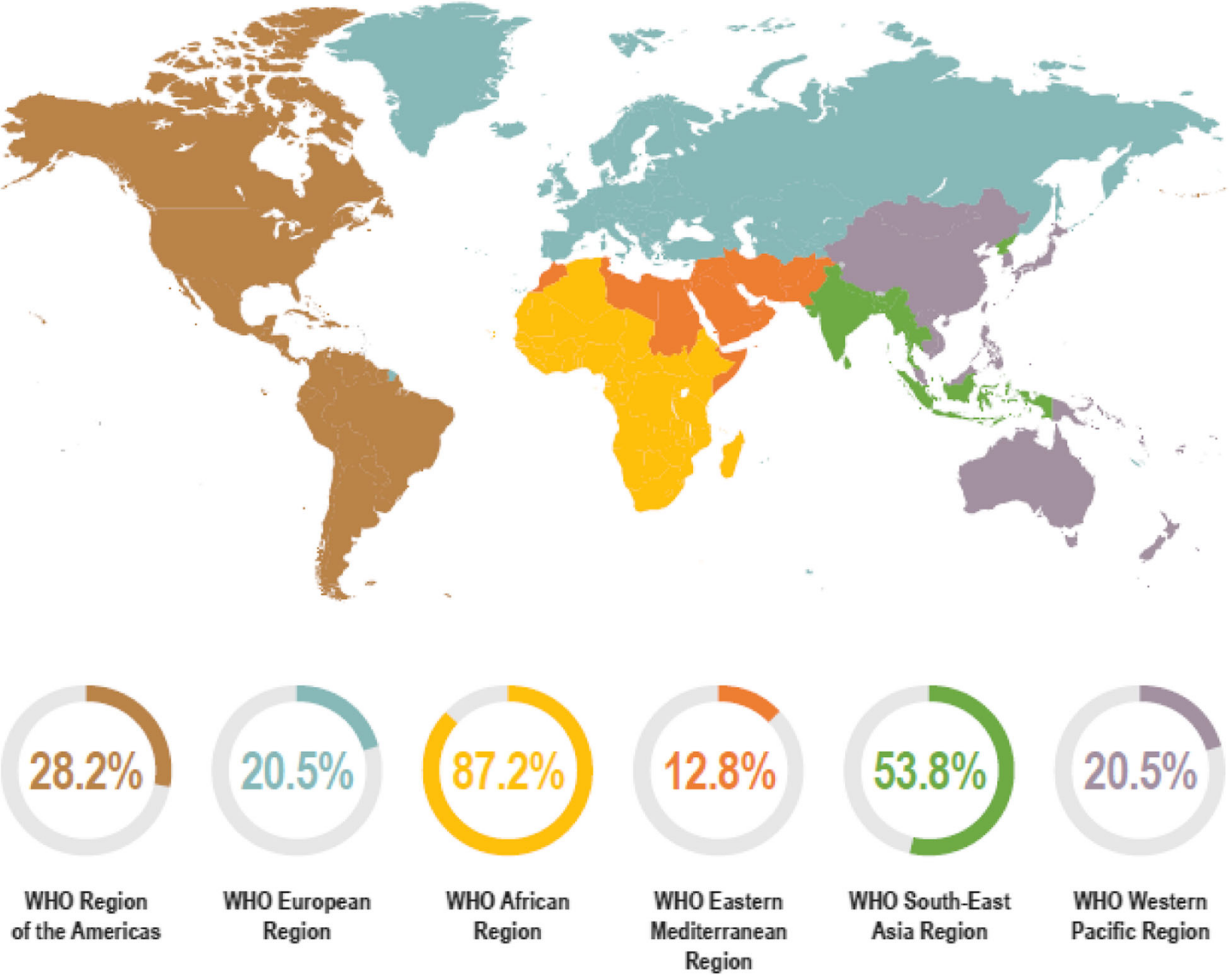
Overview of survey participant’s experience

The response rate of the expert survey was 45% (39/87). Responders listed their area(s) of expertise and they represented a diverse group (26 infectious disease clinicians; 16 public health experts; 11 other medical specializations; 11 non-malarial fever experts; 10 clinical microbiologists) and geographical areas (Fig. 2). Of the 39 survey responders, 21 (54%) agreed with the original AHP-derived list. The remaining responders made changes to the rankings (Table 1, SFI priority pathogen list). Both typhoidal and non-typhoidal *Salmonella* were ranked higher by the experts compared with the data-derived approach, moving typhoidal *Salmonella* to the top-ranked pathogen, and non-typhoidal *Salmonella* from 20th to 6th on the list. Other pathogens that increased in ranking following expert input included *Streptococcus pneumoniae* (9th to 4th place), *Staphylococcus aureus* (18th to 5th place) and *Escherichia coli* (17th to 7th place). Pathogens that decreased in ranking included *Klebsiella* species (from 5th to 10th place), *Cryptococcus* species (2nd to 12th place) and *Shigella* species (7th to 14th place). *Leishmania donovani/infantum,* Group B *Streptococcus*, Lassa fever virus and *Enterococcus faecalis* were added by experts and *Proteus mirabilis*, *Citrobacter* species, Yellow Fever virus, Japanese encephalitis virus and West Nile virus were removed from the list.

### Severe fever of febrile illness without a known source “MSF-use case” specific list

The following selection of pathogens were removed to develop this use case specific list: *Plasmodium*, *Cryptococcus* spp., and *Mycobacterium tuberculosis* (reason: due to existing diagnostic availability); *Shigella* spp., influenza A and B, *Leishmania donovani/infantum* and *Neisseria meningitidis* serogroups A, B, C, W-135, Y, and X (reason: due to lack of sufficient detection in whole blood for these pathogens; meningococcal meningitis is typically diagnosed using cerebrospinal fluid [CSF] and is only accompanied by detectable bacteremia in approximately 20% of cases); and *Mycobacterium avium complex* (reason: expected to be part of dedicated HIV panel). Further revisions to the general SFI priority pathogen list were conducted to define a SFWS priority pathogen list, which included the top 10 pathogens as an optimal panel to be detected in a single cartridge, and optimally > 10 pathogens, in order of priority, listed in Table 2. Further revisions prioritized community acquired infections over nosocomial pathogens. As such, *Klebsiella* spp. was moved from position 10 (Table 1) to position 12 (Table 2), directly following *Neisseria meningitidis*, because, although *Klebsiella* spp. can be community-acquired, it is more often a nosocomial pathogen. *Burkholderia pseudomallei* was moved from position 17 (Table 1) to position 9 (Table 2) as it was considered an ideal replacement for the vacant positions created by the prior re-ordering of *Neisseria meningitidis* and *Klebsiella* spp., as it is a community-acquired infection that meets the use case for the initial febrile illness cartridge. *Burkholderia pseudomallei* was selected for re-positioning instead of *Orientia tsutsugamushi* as the former was perceived as having a broader geographic presence whereas the latter is currently thought to mainly be endemic in the Asia-Pacific region and less present in Africa and Latin America. Moreover, because *Burkholderia pseudomallei* often clinically presents without localizing signs, and it has a significant mortality ratio (> 53%) [31, 32], it was positioned higher on the list than *Neisseria meningitidis* and *Klebsiella* spp.. *Coxiella burnetii*, an important community-acquired pathogen, was moved from position 23 (Table 1) to position 10 (Table 2). *Coxiella burnetii* can, when associated with severe disease, cause significant focal infections such as pneumonitis, hepatitis, and endocarditis. Thus it was moved higher on the list than *Haemophilus influenzae* (position 14) as the latter was considered to be a relatively less consequential pathogenic cause of severe disease. Finally, *Pseudomonas* spp. and *Acinetobacter baumannii* were moved from positions 18 and 20, respectively, and re-positioned above *Enterobacter* spp. (former position 21) because they are predominately nosocomial pathogens and less relevant to the use case for diagnosing hospital inpatient admission patients with a potential community-acquired febrile illness.

### Patient subpopulations and global distribution

**Fig. 3:**
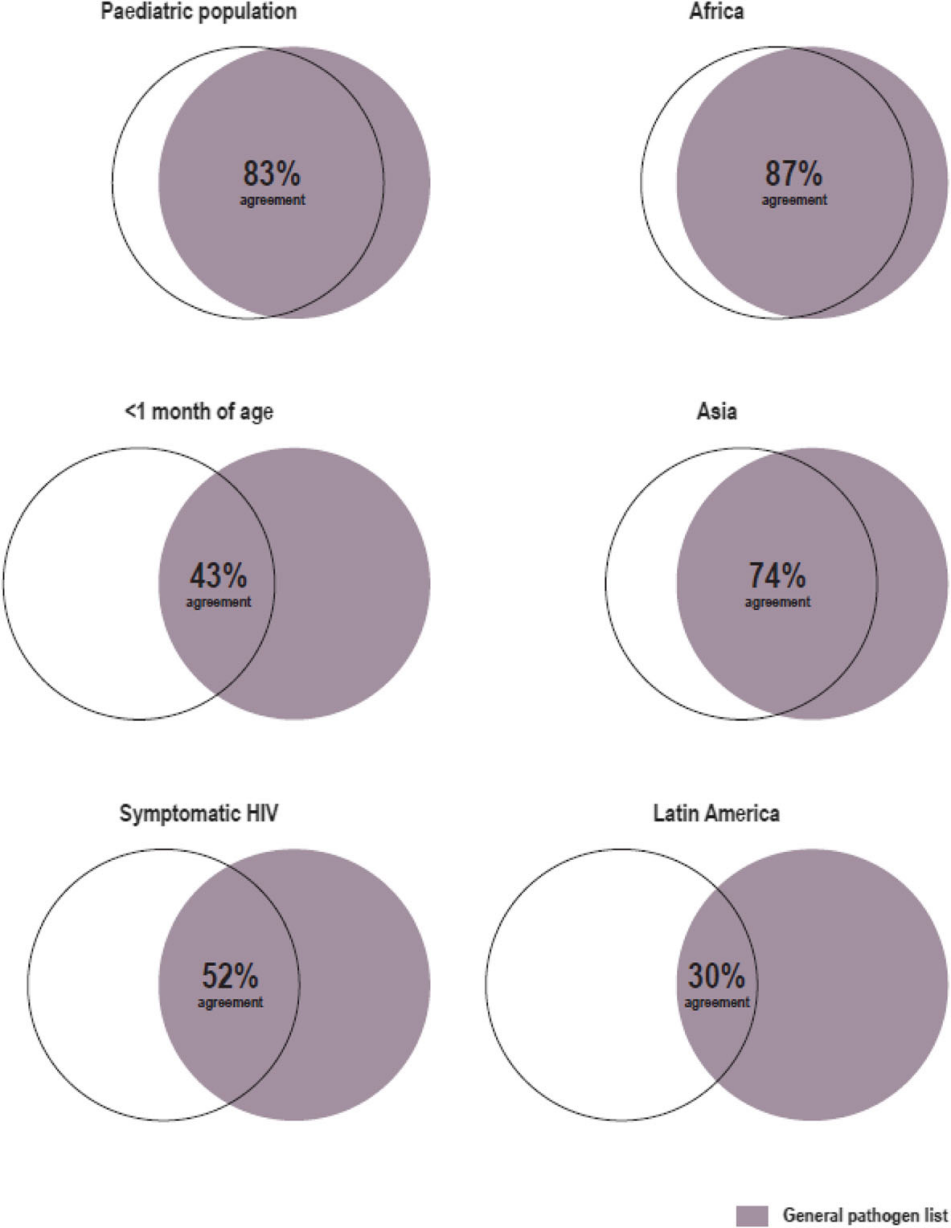
Percentage of agreement between the overall pathogen ranking for the SFWS use case and subpopulations and different regions

The kappa score to assess the level of agreement between the SFWS use case specific list showed almost perfect agreement between the combined paediatric population and the general pathogen list (percentage agreement 83% [19/23], κ = 0.82). However, in the < 1 month of age and symptomatic HIV categories, agreement was moderate (< 1 month: percentage agreement 43% [10/ 23], κ = 0.42; symptomatic HIV: 52% [12/23], κ = 0.51). The percentage of agreement for the other geographies compared with the SFWS use case specific list were as follows: Africa: 87% (20/23), κ = 0.86 (almost perfect agreement); Asia: 74% (17/23), κ = 0.73 (substantial agreement)]; Latin America: 30% (7/23), κ = 0.29 (fair agreement) (Fig. 3). Examples of published references used to determine relevancy for each pathogen to each subpopulation are shown in Additional file 3.

## Discussion

This study combined a data- and expert-driven methodology to rank pathogens responsible for SFWS in LMICs. This two-pronged approach was developed to address the lack of quality, comprehensively global and regional data, while, at the same time, not relying entirely on the potentially equally biased experience and/or opinions of clinicians. The priority pathogens identified in this study represent the basis for development and intervention prioritizations beyond the diagnostic question that initiated this work. The general SFI priority pathogen list generated by this robust two-pronged approach was then applied for further use case adaptation and demonstrates how this pathogen list could be applied to various other use case specific applications.

The top 10 of the final SFWS fever priority pathogen list before and after the stakeholder input did not include any viral pathogens and largely represents classic causes of blood stream infections and sepsis [33, 34]. Unsurprisingly, malaria, *Cryptococcus* spp. and *M. tuberculosis* were among the top 3 pathogens on both lists. This is due to the large number of infections driven by the HIV epidemic, and the associated morbidity and mortality [35, 36]. While *M. avium* complex, *Cryptococcus* spp., *Shigella* spp., *B. pseudomallei* and *O. tsutsugamushi* infections ranked high based on the available data used in the data-derived process and the subsequent AHP ranking, the consulted stakeholders deprioritized these pathogens in favour of *S. aureus*, typhoidal and non-typhoidal *Salmonella, E. coli* and *Rickettsia* spp. This de-prioritization of *B. pseudomallei* (the causative agent of meliodosis) and *O. tsutsugamushi* (the causative agent of scrub typhus) was surprising in light of recent focus on the emergence of both pathogens and their potential global impact on morbidity and mortality [31, 37–40]. However, both diseases are currently strongly associated with the Asia Pacific region, hence stakeholders with a predominant focus on Africa might have underestimated their relevance at a global level. Typhoidal and non-typhoidal *Salmonella* (NTS) were both moved to the top of the list based on stakeholder feedback, which reflects the current attention on the global agenda, especially for NTS as a co-infection with malaria and HIV [41]. With the emergence of substantial drug resistance in *S.* Typhi, good surveillance data to support interventions with the available typhoid vaccine as well as treatment strategies are crucial. It is further expected that, with the rollout of the typhoid vaccine and the resulting decreased burden of typhoidal *Salmonella*, non-typhoidal *Salmonella* will become the most frequent form of salmonellosis, supporting the observed prioritization [42–44]. Notably, *B. pseudomallei* was given specific attention in the “MSF-use case”.

Based on the sensitivity analysis, the most critical category in the AHP approach was shown to be treatment impact, which also had the highest relative weight in the original hierarchy. While the value of a pathogen’s score was modified by the perturbation added to the data in our sensitivity analysis, the relative ranking was only minutely affected, showing that the model is only marginally influenced by change in inputs, compatible with values that can be found in current literature. Changes in the severity sub-criteria were shown to impact scores to a lesser degree; however, based on our data, future efforts that aim to prioritize pathogens need to take special care in regard to those criteria.

Defining globally relevant febrile illness priorities is challenging, and even an approach that aims to address all the challenges may not capture the true needs of many geographic settings, sub-populations and use cases. A number of the surveyed experts commented that a global, context-agnostic selection of pathogens is impossible and more geographically targeted analyses were favoured. However, the kappa analysis of the SFWS use case specific list demonstrated high relevance across the various patient populations assessed, with the lowest level of agreement for < 1 month of age and symptomatic HIV patients. However, as these are specific subgroups requiring more dedicated diagnostic support and clinical attention, testing for these special patient populations will likely require their own dedicated pathogen prioritization efforts in any case and it may not be appropriate to combine their priorities with a general SFI use case. The kappa analysis comparing geographic relevance across Africa, Asia and Latin America demonstrated that a general list could be developed that is applicable for Africa and Asia, but with less relevance for Latin America. The ‘almost perfect agreements’ between lists for the paediatric and African subpopulations, and the ‘substantial agreement’ for the Asian subpopulation, suggest that, despite differences in SFI aetiology by age and geography, the application of the general fever priority pathogen list to any of these cohorts could be beneficial for improving patient clinical management outcomes. However, given only ‘fair agreement’ was achieved for the Latin American population, further targeted lists for these populations are warranted. This highlights that grouping geographies and populations together is difficult but not impossible; furthermore, to realize a commercially sustainable tool, it is important to extend the pathogen panel to the largest regional or global population possible.

Overall, while we aimed for a balanced approach, this work and the resulting general fever priority pathogen list has limitations, including its focus on SFI and then SFWS and the prescription of a specific use case to the stakeholder group. The aim was to focus attention and remove variability between patient definitions; yet, it is ultimately not possible to assess how individual stakeholders approached the use case and therefore ranked pathogens. It is clear from the literature that the aetiologies of diseases might vary between outpatients, inpatients and intensive care patients and a bias could have been introduced. While our approach was deliberately designed to compensate for the lack of epidemiological data, our work and the resulting general fever priority pathogen list might still have been affected by a bias introduced by a lack of data or/and personal perception. Stakeholder decision-making might have been influenced by a perceived over- or under-representation of a certain disease e.g. *Salmonella*. Furthermore, stakeholders pre-dominantly represented the African region and pathogens with specific relevance to the Asia Pacific or Latin America regions might have been deemphasised. Furthermore, the available pathogen prevalence data is an aggregate for very large regions of Asia, Africa and Lantin America and does not provide sufficient detail as to specific regions or account for heterogeneity in pathogen prevalence within these subregions.

Despite its many limitations, the product of this work represents a novel pathogen prioritization list using a well-recognized and established methodology. This process can help to inform prioritization efforts by global health professionals as part of diagnostic development activity, infrastructure, capacity building or research interventions. The SFWS MSF use case pathogen prioritization list was created to develop a target product profile for a multiplex/multi-analyte test that was further adapted to meet the needs of MSF settings [23, 45]. In the long-term, it is essential to ensure that additional high-quality aetiology data are established across all geographies and demographies and which include seasonal and other relevant variations. Our approach will require updates with emerging data to capture any changes in knowledge or transmission.

## Conclusion

This work highlighted, once again, the challenges of prioritizing in global health, [46, 47] but it also shows that taking a two-pronged combined approach that kept “actionability” at the centre, and that used available aetiology data and expert opinions, can result in a broadly applicable priority list of pathogens for SFWS. This broad applicability is particularly important in the context of product development, where a sufficient market size is essential to achieve a sustainable commercialized diagnostic product and ensure testing is available where it is most needed. Ultimately, without comparable, high quality data and advanced diagnostic capabilities to support diagnosis and surveillance, patient care will remain a guessing game, while it should be driven by evidence. If developed, a fever panel such as the one described here, could provide access to fit-for-purpose diagnostic testing so urgently needed to facilitate appropriate treatment and care especially at district level hospitals with no microbiology laboratories.

## Supplementary information

**Additional file 1.** AHP absolute evaluation methodology.

**Additional file 2.** Expert survey.

**Additional file 3.** Example references used to determine relevance of pathogen ranking to subpopulations and different regions.

**Additional file 4.** Introductory document for stakeholder survey outlining use-case.

## Data Availability

The datasets generated and/or analysed during the current study are available as as supplementary material.

## Abbreviations

AHP: Analytical hierarchy process
FIND: Foundation of Innovative New Diagnostics
HIV: Human immunodeficiency virus
LMICs: Low- and middle-income countries
MAPDx: Multi-pathogen and multi-analyte diagnostic platform
MSF: Médecins Sans Frontières
NTS: Non-typhoidal *Salmonella*
RDT: Rapid diagnostic test
RLS: Resource-limited settings
SFI: Severe febrile illness
SFWS: Severe febrile illness without a known source
WHO: World Health Organization
