# Supplementary material for: Prioritising pathogens for the management of severe febrile patients to improve clinical care in low- and middle-income countries
Source: BMC Infect Dis. 2020 Feb 10;20:117. doi: 10.1186/s12879-020-4834-1 (PMC7011354; doi:10.1186/s12879-020-4834-1)
Supplement: Supplementary file 2 — Additional file 2. Expert survey. [file 12879_2020_4834_MOESM2_ESM.pdf]

Fever Pathogen Priority Survey

(untitled)

SECTION 1: Demographics

1.

What is the title of your current position?

*

2.

What is the name of the organization that you currently and/or primarily work for?

*

3. Overall, how many years of work and/or research experience do you have with regard to any type of fever-causing diseases in the global health context? Select all that apply.

	Not Applicable	1-5 years	5-10 years	> 10 years
Clinical Work	○	○	○	○
Research	○	○	○	○
Enter another option	○	○	○	○

4. What is your medical and/or research specialty? Select all that apply. *

- [ ] Pediatrician
- [ ] Infectious disease specialist (adult)
- [ ] Infectious disease specialist (peds)
- [ ] Clinical microbiologist
- [ ] Internal medicine
- [ ] Surgery
- [ ] Public health
- [ ] Diagnostics
- [ ] Non-malarial fevers
- [ ] Other - Write In

5. What are the geographic regions of experience that you have in low resource settings? (select all that apply) *

- [ ] African Region
- [ ] Region of the Americas
- [ ] South-East Asia Region
- [ ] European Region
- [ ] Eastern Mediterranean Region
- [ ] Western Pacific Region
- [ ] Other - Write In

(untitled)

SECTION 2: Pathogen Prioritization

The TPP describes the test as being used "in the context of infectious diseases, intended for individual patient management for patients presenting with symptoms consistent with severe febrile illness without a known source (SFWS)." Note, in the envisioned context, SFWS is defined as a case for which "no diagnosis has been made after adequate history and clinical examination, available lab tests (e.g. malaria rapid test), and imaging services. It might include patients with a focus such as pneumonia, but without etiological diagnoses, or patients with no response to empiric antibiotics."

6.

Please rate your level of familiarity with the case definition and use case for this survey.

*

Not Familiar	Somewhat Familiar	Familiar	Very Familiar	Expert
○	○	○	○	○

Additional methodological details:
We used a data-derived list of fever pathogens in LMICs from a published systematic review (Prasad et al. 2015). To rank the pathogens, an analytical hierarchy process (AHP) was used, similar to other pathogen prioritization processes performed in the past (Kadohira et al. 2015, Taconelli et al. 2017). The AHP consisted of 5 categories (annual cases, severity, morbidity, patient impact, public health impact) and the corresponding values were identified in peer-reviewed journals or in MSF internal surveys (patient and public health impact), as appropriate. Weighted categories were defined based on a pairwise comparison performed by nine MSF experts and pathogens were subsequently ranked according to their weighted score, the final data-derived list in rank order is shown below. Of note, the list has been restricted to pathogens that can be detected using blood specimens.

Rank	Pathogen
1	*Plasmodium falciparum*<br>Non *Plasmodium falciparum*
2	*Cryptococcus spp**
3	*Mycobacteria tubercolosis*
4	Mycobacterium avium complex (MAC)
5	*Klebsiella spp*
6	*Neisseria meningitidis*
7	*Shigella spp*
8	*Burkholderia pseudomallei*
9	*Streptococcus pneuomiae*
10	*Orientia tsutsugamushi*
11	Typhoidal salmonella
12	*Haemophilus infuenzae*
13	Pseudomonas spp
14	Acinetobacter baumannii
15	Rickettsial spp
16	Leptospira spp
17	*Escherichia coli*
18	*Staphylococcus aureus*
19	*Brucella spp*
20	Non-typhoidal salmonella
21	*Histoplasma capsulatum*
22	*Coxiella burnetii*
23	*Proteus mirabilis*
24	Enterobacter spp
25	Citrobacter spp
26	Influenza virus
27	*Borrelia recurrentis*
28	Japanese encephalitis virus
29	Yellow fever virus
30	West Nile virus
31	Dengue virus
32	Chikungunya virus

*Spp. = species.

7. As outlined in the introduction to the survey, the objective of this survey is to identify a **globally relevant** priority pathogen list for a test for SFWS intended for use in **general patient populations** for testing with a **single blood specimen** for **individual patient management.** The test will detect a minimum of 6 pathogens, and ideally > 20 in a single cartridge.

Keeping the use case in mind, please reorder the entire list of pathogens below to the best of your ability in descending level of importance (i.e., most important in position 1, and lowest importance in position 20). *

Drag items from the left-hand list into the right-hand list to order them.

- Plasmodium spp
- Cryptococcus spp
- Mycobacteria tubercolosis
- Mycobacterium avium complex (MAC)
- Klebsiella spp
- Neisseria meningitidis (serogroups A, B, C, W-135, Y, and X)
- Shigella spp
- Burkholderia pseudomallei
- Streptococcus pneuomiae
- Orientia tsutsugamushi
- Typhoidal salmonella

- Haemophilus influenzae
- Pseudomonas spp
- Acinetobacter baumannii
- Rickettsial spp
- Leptospira spp.
- Escherichia coli
- Staphylococcus aureus
- Brucella spp
- Non-typhoidal salmonella

LOGIC Show/hide trigger exists.

8. Do you think any additions are required to the above data derived pathogen list? *

- Yes
- No

LOGIC Hidden unless: #8 Question "Do you think any additions are required to the above data derived pathogen list?" is one of the following answers ("No")

9. Your feedback is tremendously helpful to inform this pathogen prioritization process.

Are there any further comments you would like to provide at this time?

LOGIC Hidden unless: #8 Question "Do you think any additions are required to the above data derived pathogen list?" is one of the following answers ("Yes")

10. We acknowledge that the above list might not include pathogens that are important in your region or in your practice. What if any of the following pathogens should replace those on the data-derived list you just rank ordered? *

- Histoplasma capsulatum
- Coxiella burnetii
- Proteus mirabilis
- Enterobacter spp
- Citrobacter spp
- Influenza virus A, B, and C
- Borrelia recurrentis
- Japanese encephalitis virus
- Yellow fever virus
- West Nile virus
- Dengue virus 1, 2, and 3
- Chikungunya virus
- Lassa fever
- another option not listed here

Top choice to include in the data driven list

LOGIC Show/hide trigger exists. Hidden unless: Question "Top choice to include in the data driven list" is one of the following answers ("another option not listed here")

11. If you answered another option, what pathogen do you think should be added? *

12. Please provide an explanation and/or peer-reviewed evidence to support your suggested addition(s) to the pathogen list. *

LOGIC Hidden unless: #11 Question "If you answered another option, what pathogen do you think should be added?"

13. An addition of a pathogen needs to be supported by evidence (peer-reviewed publications; clinical case records or unpublished data for review). Please add any evidence such as:

Personal experience, data can be provided from one or more settings
Organizational experience, data can be provided from one or more settings
Published evidence from multiple places

Browse...

LOGIC Hidden unless: #11 Question "If you answered another option, what pathogen do you think should be added?"

14. Please consider providing an email address so we may contact you to discuss the additional pathogens you have recommended. Note your survey response will be not shared externally and your contact information will not be shared.

LOGIC Show/hide trigger exists. Hidden unless: Question "Top choice to include in the data driven list"

15. This additional pathogen should be in the *

- Top 6
- Top 15
- No opinion

LOGIC Show/hide trigger exists. Hidden unless: #15 Question "This additional pathogen should be in the"

16.

What should be removed from the list to accommodate this substitution?

*

Plasmodium spp
Cryptococcus spp
Mycobacteria tubercolosis
Mycobacterium avium complex (MAC)
Klebsiella spp
Neisseria meningitidis (serogroups A, B, C, W-135, Y, and X)
Shigella spp
Burkholderia pseudomallei
Streptococcus pneuomiae
Orientia tsutsugamushi
Typhoidal salmonella
Haemophilus influenzae
Pseudomonas spp
Acinetobacter baumannii
Rickettsial spp
Leptospira spp.
Escherichia coli
Staphylococcus aureus
Brucella spp
Non-typhoidal salmonella

LOGIC Show/hide trigger exists. Hidden unless: #16 Question "
What should be removed from the list to accommodate this substitution?
"

17.

Would you like to add any additional pathogens to the list?

*

- Yes
- No
- No opinion

LOGIC Hidden unless: #17 Question "
Would you like to add any additional pathogens to the list?

" is one of the following answers ("Yes")

18. What if any of the following pathogens should replace those on the data-derived list? *

- Histoplasma capsulatum
- Coxiella burnetii
- Proteus mirabilis
- Enterobacter spp
- Citrobacter spp
- Influenza virus A, B, and C
- Borrelia recurrentis
- Japanese encephalitis virus
- Yellow fever virus
- West Nile virus
- Dengue virus 1, 2, and 3
- Chikungunya virus
- Lassa fever
- another option not listed here

additional pathogen change to the data derived list

LOGIC Show/hide trigger exists. Hidden unless: Question "additional pathogen change to the data derived list" is one of the following answers ("another option not listed here")

19. If you answered another option, what pathogen do you think should be added? *

LOGIC Hidden unless: #19 Question "If you answered another option, what pathogen do you think should be added?"

20. Please provide an explanation and/or peer-reviewed evidence to support your suggested addition(s) to the pathogen list. *

LOGIC Hidden unless: #19 Question "If you answered another option, what pathogen do you think should be added?"

21. An addition of a pathogen needs to be supported by evidence (peer-reviewed publications; clinical case records or unpublished data for review). Please add any evidence such as:

Personal experience, data can be provided from one or more settings

Organizational experience, data can be provided from one or more settings

Published evidence from multiple places

Browse...

LOGIC Hidden unless: #19 Question "If you answered another option, what pathogen do you think should be added?"

22. Please consider providing an email address so we may contact you to discuss the additional pathogens you have recommended. Note your survey response will be not shared externally and your contact information will not be shared.

LOGIC Show/hide trigger exists. Hidden unless: #17 Question "
Would you like to add any additional pathogens to the list?
" is one of the following answers ("Yes")

23. This additional pathogen should be in the *

- Top 6
- Top 15
- No opinion

LOGIC Hidden unless: #23 Question "This additional pathogen should be in the"

24.

What should be removed from the list to accommodate this substitution?

*

Plasmodium spp
Cryptococcus spp
Mycobacteria tubercolosis
Mycobacterium avium complex (MAC)
Klebsiella spp
Neisseria meningitidis (serogroups A, B, C, W-135, Y, and X)
Shigella spp
Burkholderia pseudomallei
Streptococcus pneuomiae
Orientia tsutsugamushi
Typhoidal salmonella
Haemophilus influenzae
Pseudomonas spp
Acinetobacter baumannii
Rickettsial spp
Leptospira spp.
Escherichia coli
Staphylococcus aureus
Brucella spp
Non-typhoidal salmonella

LOGIC Hidden unless: #17 Question "
Would you like to add any additional pathogens to the list?
" is one of the following answers ("Yes","No","No opinion")

25. Your feedback is tremendously helpful to inform this pathogen prioritization process.

Are there any further comments you would like to provide at this time?

Thank You!

Thank you for taking our survey. Your response is very important to us.
