# Supplementary material for: Prioritising pathogens for the management of severe febrile patients to improve clinical care in low- and middle-income countries
Source: BMC Infect Dis. 2020 Feb 10;20:117. doi: 10.1186/s12879-020-4834-1 (PMC7011354; doi:10.1186/s12879-020-4834-1)
Supplement: Supplementary file 3 — Additional file 3. Example references used to determine relevance of pathogen ranking to subpopulations and different regions. [file 12879_2020_4834_MOESM3_ESM.docx]

| **Pathogen** | **Paediatrics** | **Symptomatic HIV infection** | **Africa** | **Asia** | **Latin America** |
| --- | --- | --- | --- | --- | --- |
| Typhoidal salmonella | 1,5,8,10,11,12,13,15,18, 19,20,21,23 | 1,2 | 1,2,4,14,15,17,21,23, 27,29 | 2,5,6,7,17, 26 |  |
| *Streptococcus pneumoniae* | 1,4,5,10,11,18,23,26 | 1,2,3,5,23 | 1,2,4,17,21,23,27,28,29 | 2,5,6,17,26 | 30,36 |
| *Staphylococcus aureus* | 1,4,10,11,18,19,23,26 | 1,2,3 | 1,2,4,17,21,23,27,28,29 | 1,2,5,6,17,26 | 30,31,33,34 |
| Non-typhoidal salmonella | 1,8,10,11,18,19,20,21,23,26, 28 | 1,2,3,5,23, 28 | 1,2,4,14,17,21,23,28,29 | 1,2,5,6,17,26 |  |
| *Escherichia coli* | 1,10,11,18,19,20,26 | 1,2,3 | 1,2,14,15,17, 21,23,27,29 | 1,2,5,6,17,26 | 30,33 |
| *Rickettsia* spp | 12,13,16,18,26 |  | 2,4,16 | 6,7,9,26 |  |
| *Leptospira* spp | 13,16,18,26 |  | 2,4,15,16 | 2,5,6,7,26 |  |
| *Brucella* spp | 12,16 |  | 2,4,15,16,23 |  |  |
| *Burkholderia pseudomallei* |  |  |  | 7,26 |  |
| *Coxiella burnetii* | 16,18 |  | 2,4,16 |  |  |
| *Neisseria meningitidis** | 11,18,23 | 4 | 4,17,23,29 | 17 |  |
| *Klebsiella* spp | 1,10,11,19,20,23,26 | 1 | 1,2,17,23,27,29 | 1,2,5,17,26 | 30,33 |
| *Orientia tsutsugamushi* | 26 |  |  | 2,7,9,26 |  |
| *Haemophilus influenzae* | 1,5,10,11, 13,18,23,26 | 1 | 15,17,27,29 | 5,6,17,26 |  |
| Dengue virus† | 13,26 |  | 2 | 2,6,7,9,13,26 |  |
| *Histoplasma capsulatum* |  | 4,5 | 2 |  |  |
| Lassa Fever virus |  |  | 37 |  |  |
| *Enterococcus faecalis* | 1,10 |  |  |  |  |
| *Borrelia recurrentis* |  |  | 2,4 |  |  |
| Chikungunya virus | 13,16 |  | 2,16 | 13 |  |
| *Pseudomonas* spp | 1,11,18,19,20,23 | 1,3 | 23,29 | 1,5,26 | 30,33 |
| *Acinetobacter baumannii* | 10,11,18,19,20,23,26 | 2 | 2,23,27 | 26 | 30 |
| *Enterobacter* spp | 10,20,23,26 | 1 | 4,23 | 1,26 | 32 |

spp, species. *Serogroups A, B, C, W-135, Y, and X; †Types 1, 2 and 3

**Reference list:**

1. Huson MAM, Stolp SM, Van der Poll T, Grobusch MP. Community-Acquired Bacterial Bloodstream Infections in HIV-Infected Patients: A Systematic Review. *Clinical Infectious Diseases*. 2014;58(1):79–92.
2. Prasad N, Murdock DR, Reyburn H, Crump JA. Etiology of Severe fever without a source (SFWSI) Severe fever without a source (SFWSI) in Low- and Middle-Income Countries: A Systematic Review. *PLoS One*. June 30, 2015. DOI:10.1371/journal.pone.0127962.
3. Taramasso L, Tatarelli P, Di Biagio A. Bloodstream infections in HIV-infected patients. Virulence. 2016, VOL. 7, NO. 3, 320–328. <http://dx.doi.org/10.1080/21505594.2016.1158359>.
4. Maze MJ, Bassat Q, Feasey NA, et al. The epidemiology of febrile illness in sub-Saharan Africa: implications for diagnosis and management. *Clinical Microbiology and Infection.* DOI: <https://doi.org/10.1016/j.cmi.2018.02.011>.
5. Deen J, von Seidlein L, Andersen F, et al. Community-acquired bacterial bloodstream infections in developing countries in south and southeast Asia: a systematic review. Lancet Infectious Diseases. 2012;12: 480–87.
6. Susilawati TN and McBride WJH. Acute Undifferentiated Fever in Asia: A Review of the Literature. *Southeast Asian Journal of Tropical Medicine and Public Health*. Vol 45 No.3 May 2014.
7. Acestor N, Cooksey R, Newton PN, et al. Mapping the Aetiology of Non-Malarial Febrile Illness in Southeast Asia through a Systematic Review—Terra Incognita Impairing Treatment Policies. *PLoS One.* September 2012. Volume 7, Issue 9. E44269.
8. Britto C, Pollard AJ, Voysey M, Blohmke CJ. An Appraisal of the Clinical Features of Pediatric Enteric Fever: Systematic Review and Meta-analysis of the Age-Stratified Disease Occurrence. *Clinical Infectious Diseases*. 2017;64(11):1604–11.
9. Dalrymple U, Cameron E, Bhatt S, et al. Quantifying the contribution of Plasmodium falciparum malaria to febrile illness amongst African children. *eLife*. 2017;6:e29198. DOI: <https://doi.org/10.7554/eLife.29198>.
10. Williams PCM, Isaacs D, Berkley JA. Antimicrobial resistance among children in sub-Saharan Africa. *Lancet Infectious Diseases*. 2018;18: e33–44.
11. Berkley JA, Lowe BS, Mwangi I, et al. Bacteremia among Children Admitted to a Rural Hospital in Kenya. January 6, 2005. *The New England Journal of Medicine*. 352;1.
12. Chow A and Robinson JL. Fever of unknown origin in children: a systematic review. *World Journal of Pediatrics*. 2011;7(1):5-10.
13. Capeding MR, Chua MN, Hadinegoro SR, et al. Dengue and Other Common Causes of Acute Febrile Illness in Asia: An Active Surveillance Study in Children. *PLoS Neglected Tropical Diseases*. July 2013. Volume 7, Issue 7. e2331.
14. Chattaway MA, Aboderin AO, Fashae K, et al. Fluoroquinolone-Resistant Enteric Bacteria in Sub-Saharan Africa: Clones, Implications and Research Needs. *Frontiers in Microbiology*. 22 April 2016. <https://doi.org/10.3389/fmicb.2016.00558>.
15. Crump JA, Youssef FG, Luby SP, et al. Estimating the Incidence of Typhoid Fever and Other Febrile Illnesses in Developing Countries. *Emerging Infectious Diseases.* Vol. 9, No. 5, May 2003.
16. Crump JA, Morrissey AB, Nicholson WL, et al. Etiology of Severe Non-malaria Febrile Illness in Northern Tanzania: A Prospective Cohort Study. *PLoS Neglected Tropical Diseases.* July 2013. Volume 7, Issue 7. e2324.
17. Ashley EA, Lubell Y, White NJ, Turner P. Antimicrobial susceptibility of bacterial isolates from community acquired infections in Sub-Saharan Africa and Asian low and middle income countries. *Tropical Medicine and International Health*. Volume 16 no 9 pp 1167–1179 September 2011. doi:10.1111/j.1365-3156.2011.02822.x.
18. D’Acremont V, Kilowoko M, Kyungu E, et al. Beyond Malaria — Causes of Fever in Outpatient Tanzanian Children. *The New England Journal of Medicine.* February 27, 2014. 370;9.
19. Huynh B, Padget M, Garin B, et al. Burden of bacterial resistance among neonatal infections in low income countries: how convincing is the epidemiological evidence? *BMC Infectious Diseases.* 2015. 15:127. DOI 10.1186/s12879-015-0843-x.
20. Le Doare K, Bielicki J, Heath PT, Sharland M. Systematic Review of Antibiotic Resistance Rates Among Gram-Negative Bacteria in Children With Sepsis in Resource-Limited Countries. *Journal of the Pediatric Infectious Diseases Society*. pp. 1–10, 2014. DOI:10.1093/jpids/piu014.
21. Marks F, von Kalckreuth V, Aaby P et al. Incidence of invasive salmonella disease in sub-Saharan Africa: a multicentre population-based surveillance study. *The Lancet*. Vol 5 March 2017. e310.
22. Prasad N, Sharples KJ, Murdoch DR, Crump JA. Community Prevalence of Fever and Relationship with Malaria among Infants and Children in Low-Resource Areas. *Am. J. Trop. Med. Hyg*. 93(1), 2015, pp. 178–180. doi:10.4269/ajtmh.14-0646.
23. Reddy EA, Shaw AV, Crump JA. Community-acquired bloodstream infections in Africa: a systematic review and meta-analysis. *Lancet Infect Dis*. 2010;10: 417–32.
24. Reyburn H, Mbatia R, Drakeley C, et al. Overdiagnosis of malaria in patients with Severe fever without a source (SFWSI) Severe fever without a source (SFWSI) in Tanzania: a prospective study. November 2004. *British Medical Journal*. doi:10.1136/bmj.38251.658229.55.
25. Snow RW, Sartorius B, Kyalo D, et al. The prevalence of Plasmodium falciparum in sub-Saharan Africa since 1900. *Nature*. 26 October 2017. Volume550, pages515–518. doi:10.1038/nature24059.
26. Southeast Asia Infectious Disease Clinical Research Network. Causes and outcomes of sepsis in southeast Asia: a multinational multicentre cross-sectional study. *Lancet Glob Health*. 2017;5: e157–67.
27. Tadesse BT, Ashley EA, Ongarello S. Antimicrobial resistance in Africa: a systematic review. *BMC Infectious Diseases*. 2017. 17:616. DOI 10.1186/s12879-017-2713-1.
28. Uche IV, MacLennan CA, Saul A. A Systematic Review of the Incidence, Risk Factors and Case Fatality Rates of Invasive Nontyphoidal Salmonella (iNTS) Disease in Africa (1966 to 2014). *PLoS Negl Trop Dis.* 11(1): e0005118. doi:10.1371/journal.pntd.0005118.
29. Vlieghe E, Phoba MF, Muyembe Tumfun JJ, Jacobs J. Antibiotic resistance among bacterial pathogens in Central Africa: a review of the published literature between 1955 and 2008. *International Journal of Antimicrobial Agents*. doi:10.1016/j.ijantimicag.2009.04.015.
30. Sader HS, Jones RN, Andrade-Baiocchi S, Biedenbach DJ. Four-year evaluation of frequency of occurrence and antimicrobial susceptibility patterns of bacteria from bloodstream infections in Latin American medical centers. [*Diagnostic Microbiology and Infectious Disease*](https://www.sciencedirect.com/science/journal/07328893)*.* November 2002. [Volume 44, Issue 3](https://www.sciencedirect.com/science/journal/07328893/44/3), Pages 273-280.
31. Seas C, Garcia C, Salles MJ, et al. Staphylococcus aureus bloodstream infections in Latin America: results of a multinational prospective cohort study. Journal of Antimicrobial Chemotherapy, Volume 73, Issue 1, 1 January 2018, Pages 212-222, <https://doi.org/10.1093/jac/dkx350>.
32. Villegas MV, Pallares CJ, Escandon-Vargas K, et al. Characterization and Clinical Impact of Bloodstream Infection Caused by Carbapenemase-Producing Enterobacteriaceae in Seven Latin American Countries. *PLoS ONE*. 11(4): e0154092. doi:10.1371/journal.pone.0154092.
33. Sader HS, Pfaller MA, Jones RN, et al. Bacterial Pathogens Isolated from Patients with Bloodstream Infections in Latin America, 1997: Frequency of Occurrence and Antimicrobial Susceptibility Patterns from the SENTRY Antimicrobial Surveillance Program. [*Braz J Infect Dis.*](https://www.ncbi.nlm.nih.gov/pubmed/11097713?dopt=Citation) 1999 Jun;3(3):97-110.
34. Diekema DJ, Pfaller MA, Schmitz FJ, et al. Survey of Infections Due to Staphylococcus Species: Frequency of Occurrence and Antimicrobial Susceptibility of Isolates Collected in the United States, Canada, Latin America, Europe, and the Western Pacific Region for the SENTRY Antimicrobial Surveillance Program, 1997–1999. *Clinical Infectious Diseases.* 15 May 2001. Volume 32, Issue Supplement_2, 15 May 2001, Pages S114–S132, <https://doi.org/10.1086/320184>.
35. Bardach A, Ciapponi A, Rey-Ares L, et al. Epidemiology of Malaria in Latin America and the Caribbean from 1990 to 2009: Systematic Review and Meta-Analysis. *Value in Health Regional Issues.* 2015. 8C; 69-79. <http://dx.doi.org/10.1016/j.vhri.2015.05.002>.
36. Castanheira M, Gales AC, Mendes RE, et al. Antimicrobial susceptibility of Streptococcus pneumoniae in Latin America: results from five years of the SENTRY Antimicrobial Surveillance Program. *Clin Microbiol Infect*. 2004; 10: 645–651. 10.1046/j.1469-0691.2004.00872.x.
37. World Health Organization. Lassa Fever. Available at: <http://www.who.int/emergencies/diseases/lassa-fever/en/>. [Accessed 1 October 2018].
