# Supplementary material for: Prioritising pathogens for the management of severe febrile patients to improve clinical care in low- and middle-income countries
Source: BMC Infect Dis. 2020 Feb 10;20:117. doi: 10.1186/s12879-020-4834-1 (PMC7011354; doi:10.1186/s12879-020-4834-1)
Supplement: Supplementary file 4 — Additional file 4. Introductory document for stakeholder survey outlining use-case. [file 12879_2020_4834_MOESM4_ESM.pdf]

Supplement 4

# Fever Pathogen Priority Survey

---

## Pathogen Prioritization for Severe Febrile Illness Without a Known Source

Please read this page prior to starting the survey

Introduction:
Clinicians and patients in low- and middle-income countries (LMICs) frequently lack access to reliable laboratory services, particularly outside of large population centers. When laboratory services are available, a limited test menu hinders clinical decision-making and antimicrobial stewardship, leading to empiric treatment and suboptimal patient outcomes. To revolutionize laboratory capabilities in LMIC settings, Médecins Sans Frontières (MSF) has partnered with FIND and the World Health Organization (WHO) to develop a target product profile (TPP) describing a new diagnostic platform for near-patient testing of multiple pathogens and different analytes types (MAPDx). The MAPDx would offer several advantages including: testing for multiple pathogens in a panel simultaneously rather than sequential testing; testing for multiple-analyte types to detect a pathogen along the kinetics of infection or pathogens that require different detection technologies; and a semi-open design would allow for a wide menu of assay panels. The combination of these features would result in fewer diagnostic platforms that need to be maintained and a fast, clinically useful result from a single specimen.
Because fever is one of the most common reasons for admission to hospitals in low-resource settings, the initial assay panel for MAPDx, (and the focus of the present survey), is to target severe febrile illness without a known source (SFWS). Many fever causing pathogens present with similar symptomatology, thus individuals with SFWS are severely ill but lack a clear diagnosis, making effective patient management a challenge for clinicians. Thus, the next step in this work is to define and prioritize the list of pathogens for a fever panel for use in an LMIC district-hospital setting.

Ideally, we would like to develop a variety of tests, which would target specific regions and/or patient sub-populations; however, to realize a sustainable fever test, it is important to develop a pathogen panel for the largest population possible while maintaining clinical utility. Therefore, the initial test panel for SFWS is intended for use in general patient populations for testing with a single blood specimen for individual patient management.

Methods:
To obtain a list of defined and prioritized pathogens for a fever assay cartridge, we are using a pragmatic approach combining peer-reviewed data overlaid with expert knowledge. This two-pronged quantitative and qualitative approach was designed to address the lack of comprehensive global etiology data. Following this pathogen prioritization survey aimed at general patient populations, a further internal evaluation will be performed to determine the clinical relevance of applying the MAPDx to defined and targeted patient sub-populations and specific regional geographies.

Purpose:
The objective of this survey is to obtain your expert opinion on the data-derived pathogen list.
